# Regulating Neutrophil PAD4/NOX-Dependent Cerebrovasular Thromboinflammation

**DOI:** 10.7150/ijbs.77434

**Published:** 2023-01-09

**Authors:** Junaid Ansari, Shantel A. Vital, Shreya Yadav, Felicity N. E. Gavins

**Affiliations:** 1 Department of Neurology, Louisiana State University Health Sciences Center-Shreveport, Shreveport, LA, 71130, USA.; 2 Department of Molecular & Cellular Physiology, Louisiana State University Health Sciences Center-Shreveport, Shreveport, LA, 71130, USA.; 3 Department of Life Sciences, Centre of Inflammation Research and Translational Medicine (CIRTM), Brunel University London, London. UB8 3PH, UK.

**Keywords:** Thromboinflammation, neutrophils, thrombosis, neutrophil extracellular traps, peptidyl arginine deiminase 4, NADPH oxidase

## Abstract

**Background:** Neutrophil extracellular trap (NET) production has been implicated in the pathogenesis of thromboinflammatory conditions such as Sickle Cell Disease (SCD), contributing to heightened risk for ischemic stroke. NETs are catalyzed by the enzyme Peptidyl Arginine Deiminase 4 (PAD4) and neutrophil derived reactive oxygen species (ROS), especially NADPH oxidase (NOX) which interacts with PAD4 and is therefore critical for neutrophil function. However, the role that NOX-dependent ROS and NETs play in the accelerated cerebral microvascular thrombosis associated with thromboinflammatory conditions, such as SCD, has not been fully elucidated and is the aim of this study.

**Methods:** The *in-vitro* effects of targeting PAD4 and NOX were examined using physiologically relevant NET assays with neutrophils isolated from healthy volunteers (control) and SCD patients. In addition, *in-vivo* intravascular effects of targeting PAD4 and NOX in the cerebral microcirculation of C57BL/6 and sickle transgenic mice (STM) were assessed using a photoactivation thrombosis model (light/dye) coupled with real-time fluorescence intravital microscopy.

**Results:** We found that targeting PAD4 and NOX in human neutrophils significantly inhibited ionomycin dependent H3cit^+^ neutrophils. Targeting PAD4 and NOX *in-vivo* resulted in prolonged blood flow cessation in cerebrovascular arterioles as well as venules. Moreover, we were able to replicate the effects of PAD4 and NOX targeting in a clinical model of accelerated thromboinflammation by increasing blood flow cessation times in cerebral microvessels in STM. These findings concurred with the clinical setting i.e. neutrophils isolated from SCD patients, which possessed an attenuation of H3cit^+^ neutrophil production on targeting PAD4 and NOX.

**Conclusions:** Taken together, our compelling data suggests that PAD4 and NOX play a significant role in neutrophil driven thromboinflammation. Targeting PAD4 and NOX limits pathological H3cit^+^ neutrophils, which may further explain attenuation of cerebral thrombosis. Overall, this study presents a viable pre-clinical model of prevention and management of thromboinflammatory complications such as ischemic stroke.

## Introduction

Inflammation and thrombosis are highly intertwined processes in which systemic inflammation can beget local thrombosis, and thrombosis can amplify inflammation. Inflammatory cells, such as neutrophils, monocytes and platelets, and their pro-inflammatory mediators play an important role in thrombus formation, contributing to a “thromboinflammatory” phenotype 1-3. Neutrophils are the main protagonists of immune surveillance and have decisive roles in guiding and executing pathophysiological responses by their ability of phenotypic differentiation and modification 4. Mechanistically these immune cell first responders play pivotal roles in immunity and repair, through an assortment of effector mediators including neutrophil extracellular traps (NETs), proteinases, pro-oxidant enzymes as well as reactive oxygen species (ROS), all of which engage in systematic thromboinflammatory actions by interacting with the cellular and vascular milieu (e.g. platelets and endothelium) 4-7. Many studies have showcased neutrophils as a double-edged sword with NETs revolutionizing the idea of a defense mechanism deployed by neutrophils to combat infection by trapping bacteria and mediating phagocytosis. However, excessive and uncontrolled NET formation is known to contribute to the pathogenesis of ischemic stroke 8-10, COVID-19 thrombosis 11, sepsis 12, vasculitis 13 and sickle cell disease (SCD) 14.

A critical step in NET formation is the decondensation of chromatin that occurs in the nucleus. Peptidylarginine deiminase (PAD4) is a nuclear enzyme that converts specific arginine residues to citrulline on histone tails, enabling chromatin decondensation and NET formation. This process is termed as citrullination 15. Although neutrophils express both PAD2 and PAD4, the latter is expressed in much higher levels 14, 16. Aberrant and excessive PAD4 expression is seen in a variety of chronic inflammatory diseases including rheumatoid arthritis, systemic lupus erythematosus, multiple sclerosis and vascular thrombotic diseases (deep venous thrombosis, atherothrombosis, atherosclerosis and myeloproliferative neoplasms) 16-18. Histone hypercitrullination associated with pathological NETosis 14 is characteristically known for its ability to enable thromboinflammation 14 by platelet activation and aggregation (via TLR2 and TLR4) 19. Furthermore, PAD4^-/-^ mice cannot citrullinate histones and are therefore incapable of forming NETs 20, increasing their susceptibility to infection. COVID-19 is considered to be a thromboinflammatory condition and several studies have now demonstrated higher level of NETs in COVID-19 patients 21, 22. Therefore it is also plausible that targeting PAD4 may have a potential role in SARS-CoV2 induced thromboinflammation 23, 24.

Collectively, these findings highlight the crucial role that PAD4 and NETs play in thromboinflammation. Novel evidence has also suggested the potential role of PAD2 in inflammation. However, this role appears more macrophage-dependent via METosis (a process of macrophage releasing extracellular traps) and pyroptosis (caspase 1 dependent cell death) 25. There is only very limited data on the role of PAD2 in NETosis and targeting it as a viable therapeutic option due to less expression in neutrophils compared to macrophages 26.

Both thrombus formation and its resolution may be regulated by ROS. ROS increases the expression and activation of tissue factor and the subsequent production of thrombin, in endothelial cells, monocytes and vascular smooth muscle cells, with ROS-generating gp91phox (NADPH oxidase [NOX]) enzymes being important contributors 27, 28. Neutrophils under activated states produce abundant amounts of ROS via NOX (the main ROS machinery in neutrophils) 29, which has been implicated to play a role in NET formation and release 30, 31. Despite the known involvement of neutrophil dependent PAD4 and ROS in the pathogenesis of thromboinflammatory diseases 20, 31, there is a limited understanding about their definitive role in contributing to cerebrovascular thrombosis and ischemic stroke. The aim of the present study was to assess the role that NOX-dependent ROS and NETs play in cerebral microvascular thrombosis and whether targeting PAD4/NOX affords protection against thrombotic events associated with thromboinflammatory conditions such as SCD.

## Material and Methods

### Drugs, reagents and antibodies

*In-vitro* experiments: vehicle 1X Phosphate buffered saline (PBS) (Life Technologies), GSK484 (10 μM. PAD4 inhibitor) 32, VAS3947 (5 μM. NOX inhibitor) 33 were used as pharmacological tools and neutrophils were pre-treated with these inhibitors for 15 minutes prior to stimulation. Dose of GSK484 and VAS3947 was based on previous studies and dose response (**Supplementary Figure 1**) 32, 34. NETs were induced by Ionomycin (4 μM) 14 (Life Technologies) or PMA (100 nM) 31 (Sigma-Aldrich). NET specific stains include SYTOX^TM^ Green nucleic acid stain (Life Technologies) (1 μM) (Abcam), histone H3 mouse anti-H3Cit (1:200) (Cell Signaling Technology) were used for NET detection. *In-vivo* experiments vehicle (saline), GSK484 (10 μM),^32^ VAS3947 (5 μM) 33 were used as pharmacological tools.

### Animals

Male and female control (C57BL/6) and Sickle Cell Transgenic mice (STM. Townes. Homozygous at the *Hba* locus for the hα mutation [*Hba^tm1(HBA)Tow^*] and homozygous at the *Hbb* locus for the -383 γ-β^A^ mutation [*Hbb^tm3(HBG1,HBB)Tow^*]) were purchased from Jackson Laboratory (Bar Harbor, ME, USA) 35. Townes mice were then bred on site. All mice were housed at LSU Health Sciences Center in Shreveport (LSUHSC-S). Mice were 8-10 weeks of age (5-6 mice per group). The Animal Care and Use Committee of LSUHSC-S approved experimental procedures performed on the mice. All studies were performed blinded and randomized, with a key system to identify which animal/sample had undergone which treatment and all studies complied with ARRIVE (Animal Research: Reporting In Vivo Experiments) guidelines. Furthermore, compounds administered were made by laboratory personnel other than the one performing the experiment.

### Human samples

The study was approved by the institutional review board of the LSUHSC-S (STUDY00000572 and STUDY00000261) and conducted in accordance with the Declaration of Helsinki. Blood was collected from human volunteers recruited from the LSUHSC-S after obtaining consent (25-54 years old, eight males, seven females). Blood was also obtained from SCD patients (20-36 years old, five males, nine females) upon routine clinical visits at the Feist-Weiller Cancer Center at LSUHSC-S. All SCD patients were on chronic hydroxyurea therapy and blood was obtained just before exchange transfusion. Hydroxyurea was started at 15 mg per kilogram of body weight per day and then escalated by 5 mg per kilogram every 12 weeks until the maximum tolerated dose was achieved based on peripheral blood counts. Patients were on partial exchange transfusion every two weeks. Patients with acute infection or other chronic blood borne diseases (HIV, Hepatitis B/C) were excluded from the study. Demographic and clinical characteristics of controls and SCD patients are included in **Supplemental Table 1**.

### Isolation of human neutrophils

Peripheral neutrophils were isolated from blood obtained from healthy humans and SCD patients using dextrose-histopaque separation followed by hypotonic lysis as described previously 36. After the plasma was removed, 1X PBS was layered slowly on top of the blood followed by 6% dextran (Spectrum Chemical). After mixing the layers slowly, the blood was allowed to sediment for 15-20 minutes. The pink leukocyte layer on the top was collected and carefully layered over histopaque 1077 (Sigma-Aldrich). This was followed by centrifugation at 1500 rpm for 30 minutes. The resultant pellet was resuspended in ice-cold ddH_2_o water and 10X PBS (hypotonic lysis) in 9:1 ratio to remove any contaminating erythrocytes. The solution was further centrifuged at 1000 rpm for 10 minutes. The final neutrophil containing pellet was resuspended in 1X PBS and the cells were counted by trypan blue dye exclusion using Neubauer hemocytometer. Finally, neutrophils were resuspended in DMEM with 3% fetal calf serum and kept on ice until further use. Neutrophils were routinely assessed for purity using Wright-Giemsa stain.

### Visualization and quantification of NETs

Neutrophils were seeded on poly-l-lysine coated coverslips (Discovery Labware) (100,000/well) in a 24-well plate or transferred directly to a 96-well plate and were stimulated for 3 hours at 37^o^C, 5% CO_2_. For immunocytochemistry, neutrophils were either stained with Sytox green (1µM) (cell-impermeable dye) or fixed (10% formalin), permeabilized (0.5% Triton X-100) and blocked (10% goat serum). After fixing cells were incubated with histone H3 mouse anti-H3Cit (1:200) followed by species-specific secondary antibody coupled with Alexa Fluor Dyes (1:1000, Alexa Fluor 488 goat anti-mouse) (Abcam). In the final step, DNA was stained with 4′,6-diamidino-2-phenylindole (DAPI; Invitrogen) in PBS for 10 minutes. After mounting (Fluoromont-G, Southern Biotech) the neutrophil containing coverslips on a glass slide, the images were visualized by Nikon Eclipse Ti inverted epifluorescence microscope (Minato-ku). *In-vitro* NETs were quantified by measuring the percentage of CitH3^+^ stained DNA over total number of neutrophils (DAPI stained) in a double blinded fashion. For precise measurements, morphological changes (characterized with protrusion of nucleus and chromatin outside the main neutrophil body) were considered for neutrophils to be positive for NETs (**Supplementary Figure 2**). Pictures were taken from three to four random fields with the total of at-least 50 cells per view and the average was calculated. In addition, NETs were also quantified by analyzing Sytox green intensity by plate reader (Synergy H1).

### ROS detection assay

DHR123 was used for detecting ROS production. Cells at a concentration of 200,000 per well in a 96 well plate were seeded and then treated with VAS3947 for 15 minutes before Phorbol 12-myristate 13-acetate (PMA) or ionomycin stimulation and were kept in a humidified incubator (37°C, 5% CO_2_) for 2 hours. DHR123 (25 μM) was added to the wells for 15 minutes before fluorescence reading using a plate reader (BioTek; excitation = 485 nm, emission = 525 nm.

### Thrombosis

Anesthetized mice (Ketamine: Xylazine, 1:1) were kept under the microscope after jugular vein cannulation and open window craniotomy. Thrombosis in cerebral vessels was induced using the light/dye thrombosis model 37. After 20 minutes of equilibration, 10 mg/kl of 5% FITC-dextran (150 000 MW) (Sigma-Aldrich) was injected via the femoral vein and allowed to circulate for 10 minutes. Photoactivation was initiated (excitation, 495 nm; emission, 519 nm) by exposing 100 μm of vessel length to epi-illumination with a 175-W xenon lamp (Lamda LS; Sutter) and a fluorescein filter cube (HQ-FITC; Chroma). Onset (i.e. when platelet aggregates first started to appear) and cessation (i.e. when blood flow stopped) times were recorded. 30 minutes prior to onset of thrombosis, mice were treated with vehicle (saline), GSK484 (10 μM) or VAS3947 (5 μM) (**see supplemental videos**).

### Statistical Analysis

All data was tested to follow a normal distribution using Kolmogorov-Smirnov test of normality with Dallal-Wilkinson-Lillie D'Agostino-Pearson omnibus normality test for corrected *p* value. Data that passed the normality assumption was analyzed using Student's *t*-test (two groups) or ANOVA with Bonferroni post-tests (more than two groups). Data that failed the normality assumption were analyzed using the non-parametric Mann-Whitney U test (two groups) or Kruskal-Wallis with Dunn's test (more than two groups). Analysis was performed using Graph Pad Prism 9 software (San Diego). Data are shown as mean values ± standard error of the mean (SEM). Differences were considered statistically significant at a value of *p*<0.05.

For original data, please contact felicity.gavins@brunel.ac.uk.

## Results

### PAD4 and NOX inhibition causes decrease in H3cit^+^ neutrophils

**Figure 1:**
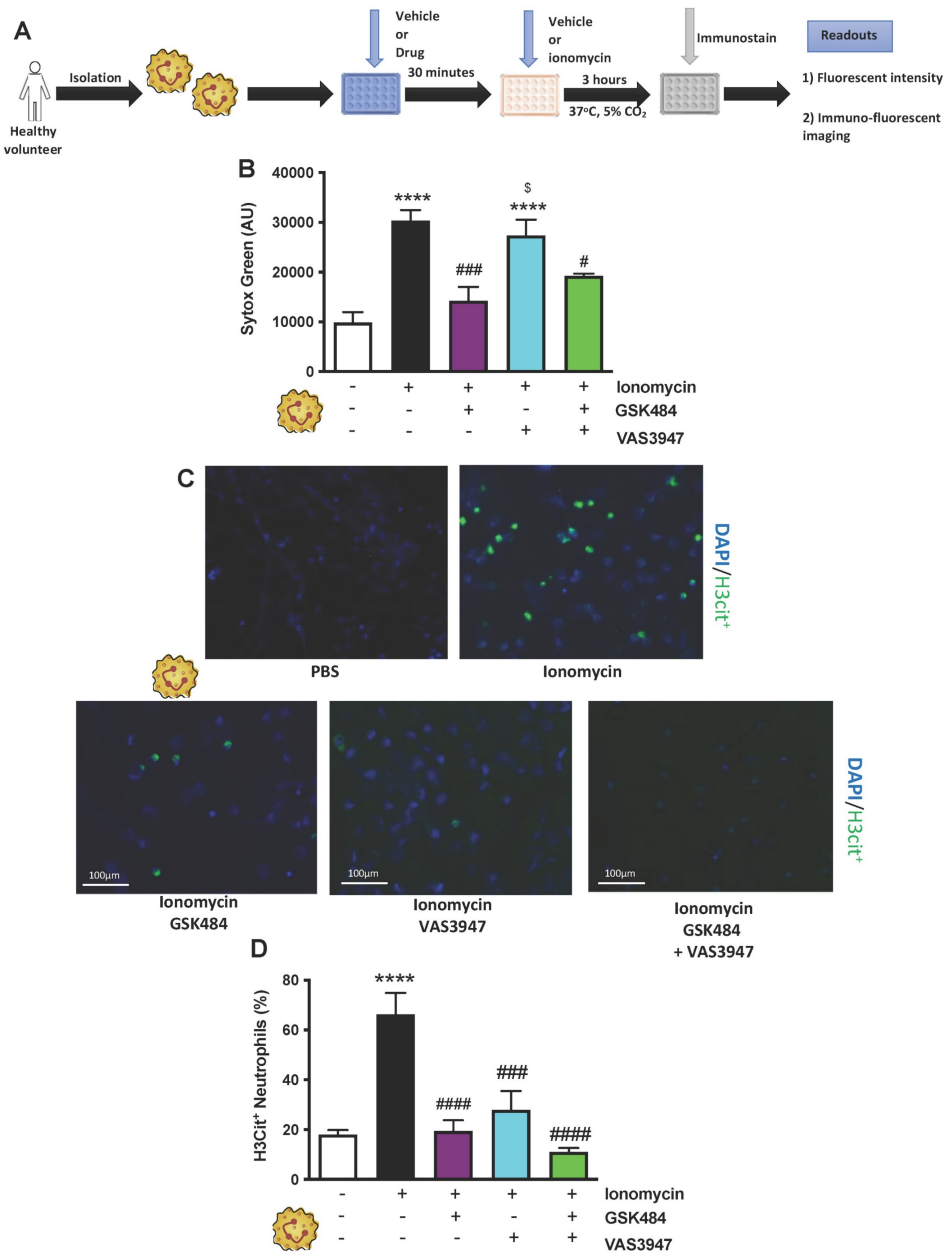
** PAD4 and NOX inhibition causes decrease in H3Cit^+^ neutrophils. (A)** Schematic of experimental design of neutrophil isolation and neutrophil extracellular trap (NET) analysis. **(B)** NETs were quantified by Sytox green intensity using a plate reader (BioTek; excitation = 485 nm, emission = 525 nm) from unstimulated and ionomycin (4 µM)-stimulated neutrophils isolated from human volunteers; unstimulated (n = 8), ionomycin stimulated (n = 8), GSK484 pre-treated ionomycin-stimulated neutrophils (n=5), VAS3947 pre-treated ionomycin-stimulated neutrophils (n=6), and GSK484 + VAS3947 pre-treated ionomycin-stimulated neutrophils (n=5). **(C+D)** In a similar manner, percentage of NETs hypercitrullinated at histone H3 (H3Cit^+^) quantified from unstimulated (n = 8), ionomycin stimulated (n = 7), GSK484 pre-treated ionomycin-stimulated neutrophils (n=5), VAS3947 pre-treated ionomycin-stimulated neutrophils (n=5), and GSK484 + VAS3947 pre-treated ionomycin-stimulated neutrophils (n=5). ^*^p<0.05, ^***^p<0.001, ^****^p<0.0001 vs. control unstimulated neutrophils.^ ####^*p<0*.0001 vs. ionomycin-stimulated neutrophils. ^$^*p<*0.05 vs. GSK484 pre-treated ionomycin-stimulated neutrophils. Graphs are expressed as mean±SEM from independent experiments. All imaging analysis was done in a double-blinded fashion.

Having previously discovered that neutrophils, and more importantly their NETs, play a key role in cerebral thrombosis 14, we wanted to further elucidate the mechanisms involved in this process. Given the key roles that neutrophil-derived ROS 31 (the main source of which is NADPH oxidase) 38 and PAD4 20 play in the NETosis-apoptosis axis, we used specific PAD4 and NOX inhibitors to target NET production. To stimulate maximal NET production, isolated neutrophils from human volunteers were treated with ionomycin (a natural calcium ionophore), with or without pre-treatment with GSK484 (PAD4 inhibitor), and VAS3947 (NOX inhibitor). (**Figure 1A**, n ≥ 5). Sytox green intensity was used for expression of neutrophil extracellular DNA 14. **Figure 1B** (n ≥ 5) shows that GSK484 significantly inhibits ionomycin-stimulated extracellular DNA production (*p*<0.001), an effect not shared by neutrophils treated with VAS3947. Furthermore, upon co-administration of GSK484+VAS3947, the PAD4 inhibitor still retained its inhibitory effects (**Figure 1B**), inferring NOX dependent ROS inhibition is not necessary for extracellular DNA production, suggesting neutrophil extracellular DNA production is NOX independent.

To specifically characterise pathological NETosis specific NET immunucytological statin Citrullinated histone-3 (H3Cit^+^. **Figure 1C**, n ≥ 5) was used 14. H3Cit^+^ is the most common NET biomarker that has been associated with experimental thrombosis 14. **Figure 1D** (n ≥ 5) shows that GSK484 (*p*<0.0001) when used alone or in combination with VAS3947 significantly reduced the percentage of ionomycin-stimulated neutrophils that were positive for H3Cit^+^ (*p*<0.0001), suggesting that targeting either PAD4 or NOX dependent ROS production has significant effect on histone hypercitrullination. Interestingly, similar to the effects observed with GSK484, VAS3947 was also able reduce the percentage of H3Cit^+^neutrophils (*p*<0.001. **Figure 1D**), an effect which was opposite to that observed with extracellular DNA production (**Figure 1B**). These results suggested that targeting both PAD4 and NOX results in suppression of ionomycin-stimulated H3Cit^+^ neutrophils. The above differences clearly suggest differences in extracellular DNA production and H3cit^+^ neutrophils production and targeting 39.

### ROS targeting is more effective and inclusive mechanism for H3Cit+ neutrophils attenuation

**Figure 2:**
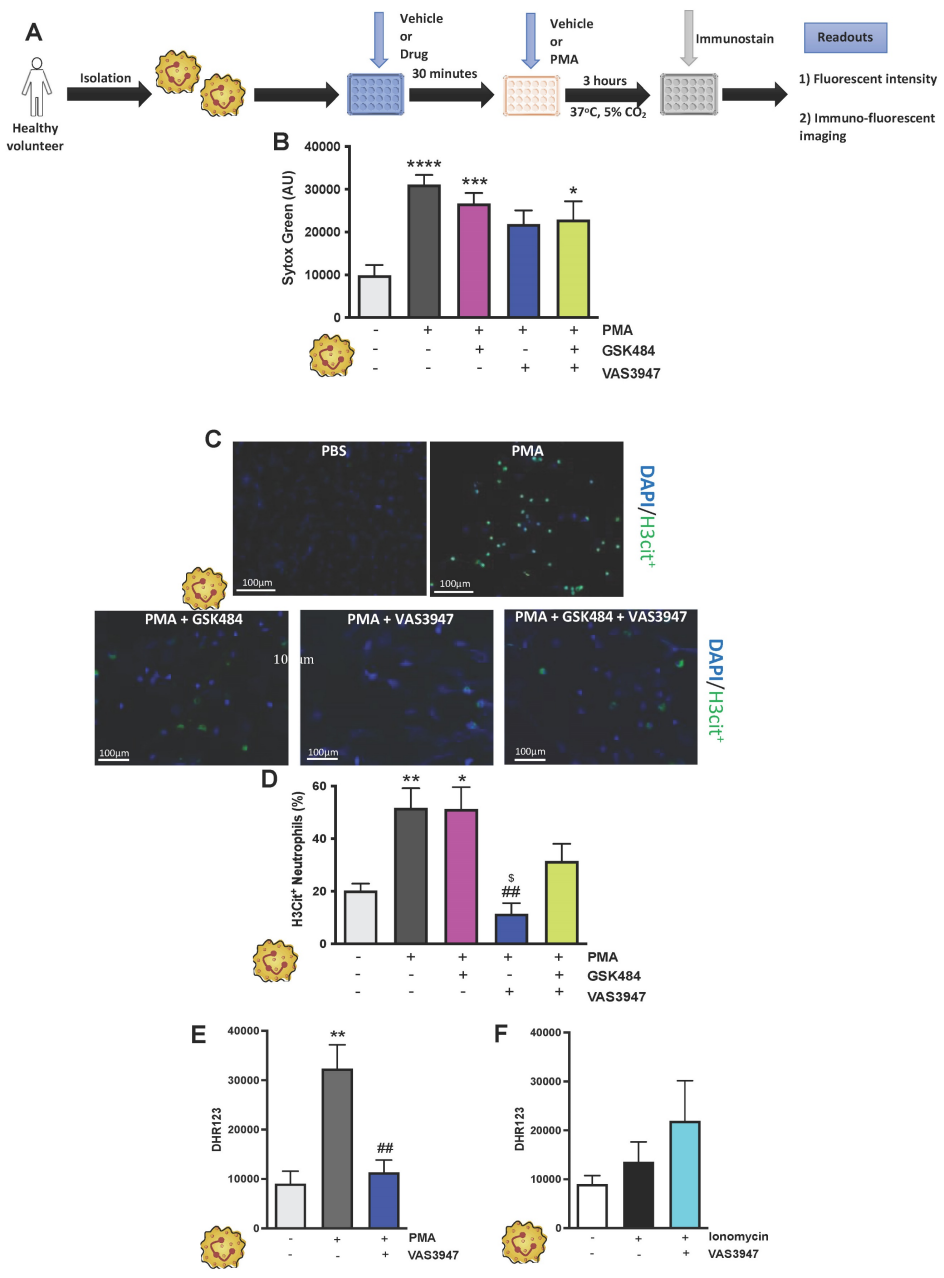
** NOX associated NET production is PAD4 independent. (A)** Schematic of experimental design of neutrophil isolation and neutrophil extracellular trap (NET) analysis. **(B)** NETs were quantified by Sytox green intensity using a plate reader (BioTek; excitation = 485 nm, emission = 525 nm) from unstimulated and PMA (100 nM)-stimulated neutrophils isolated from human volunteers; unstimulated (n = 10), PMA stimulated (n = 9), GSK484 pre-treated PMA-stimulated neutrophils (n=10), VAS3947 pre-treated PMA-stimulated neutrophils (n=5), and GSK484 + VAS3947 pre-treated PMA-stimulated neutrophils (n=5). (C) Images of NETs with merged H3Cit (green/Alexa Fluor 488), NE and nucleus (Blue/49,6-diamidino-2-phenylindole). Bars in images represent 100 mm. **(D)** In a similar manner, percentage of NETs hypercitrullinated at histone H3 (H3Cit^+^) quantified from unstimulated (n = 9), PMA-stimulated (n = 9), GSK484 pre-treated PMA-stimulated neutrophils (n=9), VAS3947 pre-treated PMA-stimulated neutrophils (n=6), and GSK484 + VAS3947 pre-treated PMA-stimulated neutrophils (n=5). **(E+F)** DHR123 production was measured using a plate reader (BioTek; excitation = 485 nm, emission = 525 nm) from **(E)** unstimulated neutrophils, PMA-stimulated neutrophils, and VAS3947 treated PMA-stimulated neutrophils (n=5 in all groups), and from **(F)** unstimulated neutrophils, ionomycin-stimulated neutrophils, and VAS3947 treated ionomycin-stimulated neutrophils (n=5 in all groups).^ *^p<0.05, ^**^p<0.01,^ ***^p<0.001, ^****^p<0.0001 vs. control unstimulated neutrophils.^ ##=^*p<*0.01 vs. PMA-stimulated neutrophils. ^$^*p<*0.05 vs. GSK484 pre-treated PMA-stimulated neutrophils. Graphs are expressed as mean±SEM from independent experiments. All imaging analysis was done in a double-blinded fashion.

It is known that different NET stimuli may have variable responses depending on the NET-associated intracellular signalling pathway engagement 38, 40. PMA induces NET via ROS production by NOX, a mechanism which is considered physiologically more reflective of inflammation 30, 41. GSK484 failed to suppress PMA dependent extracellular DNA production (**Figure 2A-C**, n ≥ 5) as well as H3Cit^+^ neutrophils (**Figure 2D**, n ≥ 5). In a similar fashion VAS3947 was unable to suppress PMA dependent extracellular DNA production further suggesting that extracellular DNA production is NOX independent (**Figure 2B**). However, VAS3947 significantly suppressed PMA dependent H3Cit^+^ neutrophils (*p*<0.001) (**Figure 2C+D**). Additionally, VAS3947 was able to inhibit PMA dependent ROS production (*p*<0.001) (**Figure 2E**, n ≥ 5). On the contrary, ionomycin was not able to increase ROS production in human neutrophils (**Figure 2F**, n ≥ 5). Finally, there was no effect of ROS production in VAS3947 treated ionomycin stimulated human neutrophils (**Figure 2F**). These findings further confirm the importance of ROS in H3Cit^+^ neutrophil production irrespective of neutrophil stimuli.

### PAD4 and NOX dependent ROS inhibition attenuate *in-vivo* cerebrovascular thrombosis

**Figure 3:**
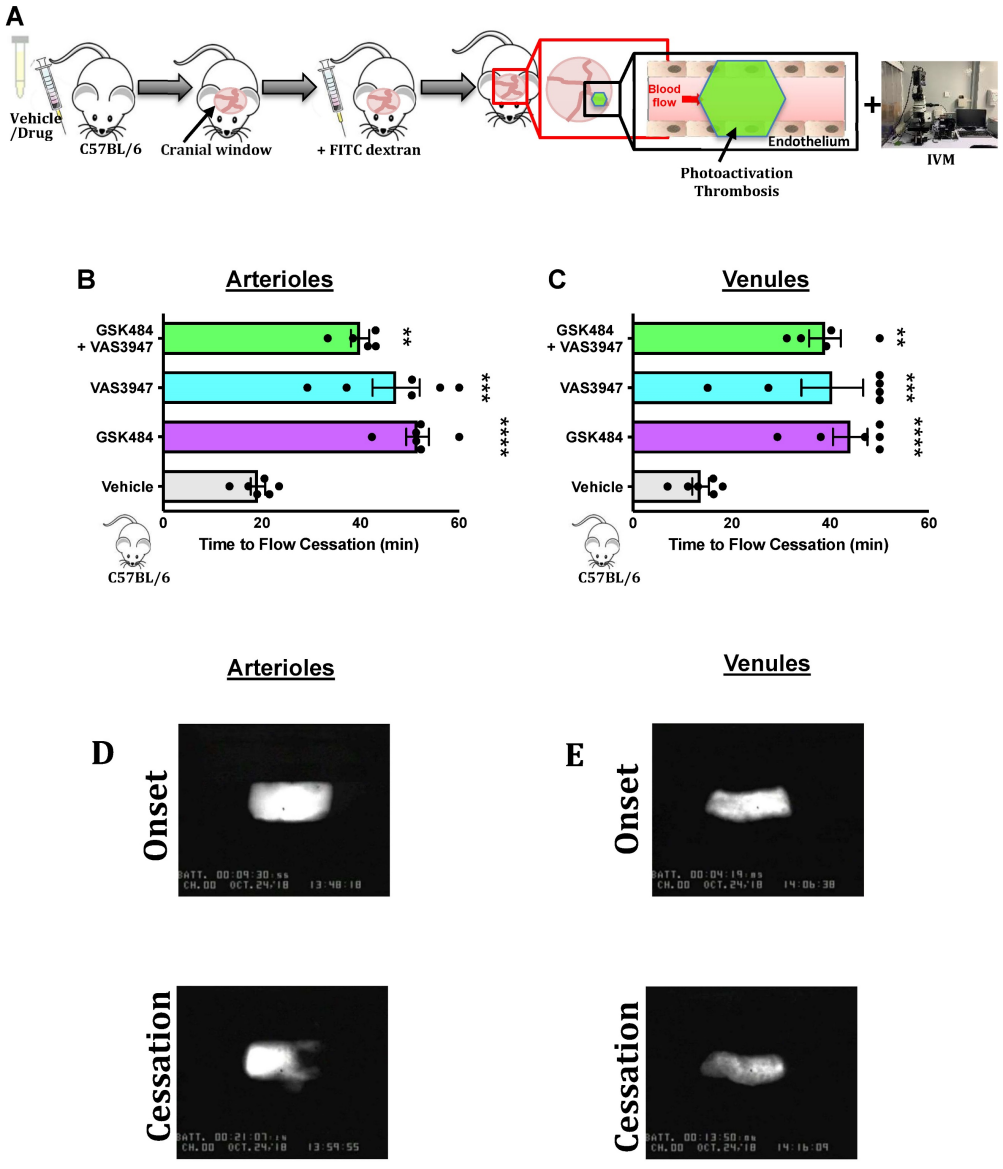
** PAD4 and NOX targeting attenuate *in-vivo* cerebrovascular thrombosis. (A)** Schematic representation of light/dye-induced thrombosis model induced with intravenous infusion of 10 mg/kg of 5% FITC-dextran followed by photoactivation of the cerebral microvessels of C57BL/6 mice. Time to flow cessation (minutes) was defined as the complete stop of blood flow for ≥ 30 seconds and was assessed in **(B)** arterioles and **(C)** venules in C57BL/6 mice treated with vehicle (saline. n=6 mice per group), GSK484 (10 μM. n=6 mice per group), VAS3847 (5 μM/ n=6 mice per group) or a combination of GSK484 + VAS3947 (n=5 mice per group). **(D-E)** Images of onset (start of platelet aggregation) and cessation (complete stop of flow for ≥30 seconds) in arterioles and venules respectively. ^**^p<0.01,^ ***^p<0.001, ^****^p<0.0001 vs. vehicle treated mice. Graphs are expressed as mean±SEM from 5-6 mice per group.

Having found that inhibition of PAD4 and ROS hold significant repressive effects on extracellular NET production as well as H3Cit^+^ neutrophils, we wanted to see the effect of such approach on vascular thrombosis using fluorescence intravital microscopy with light/dye injury model of thrombosis (**Figure 3A**) 42. Given the differences between arteriolar and venular vessels and subsequent thrombogenesis, we conducted our experiments in both cerebral arteriolar and venular vessels 37. **Figure 3B+C** (n ≥ 5) shows that both GSK484 and VAS3947, when used alone or in combination, were able to significantly prolong blood flow cessation in cerebrovascular arterioles as well as venules clearly demonstrating that targeting PAD4 and NOX production is an effective strategy to mitigate vascular thrombosis.

### PAD4 and NOX are important mediators of human thromboinflammation

**Figure 4:**
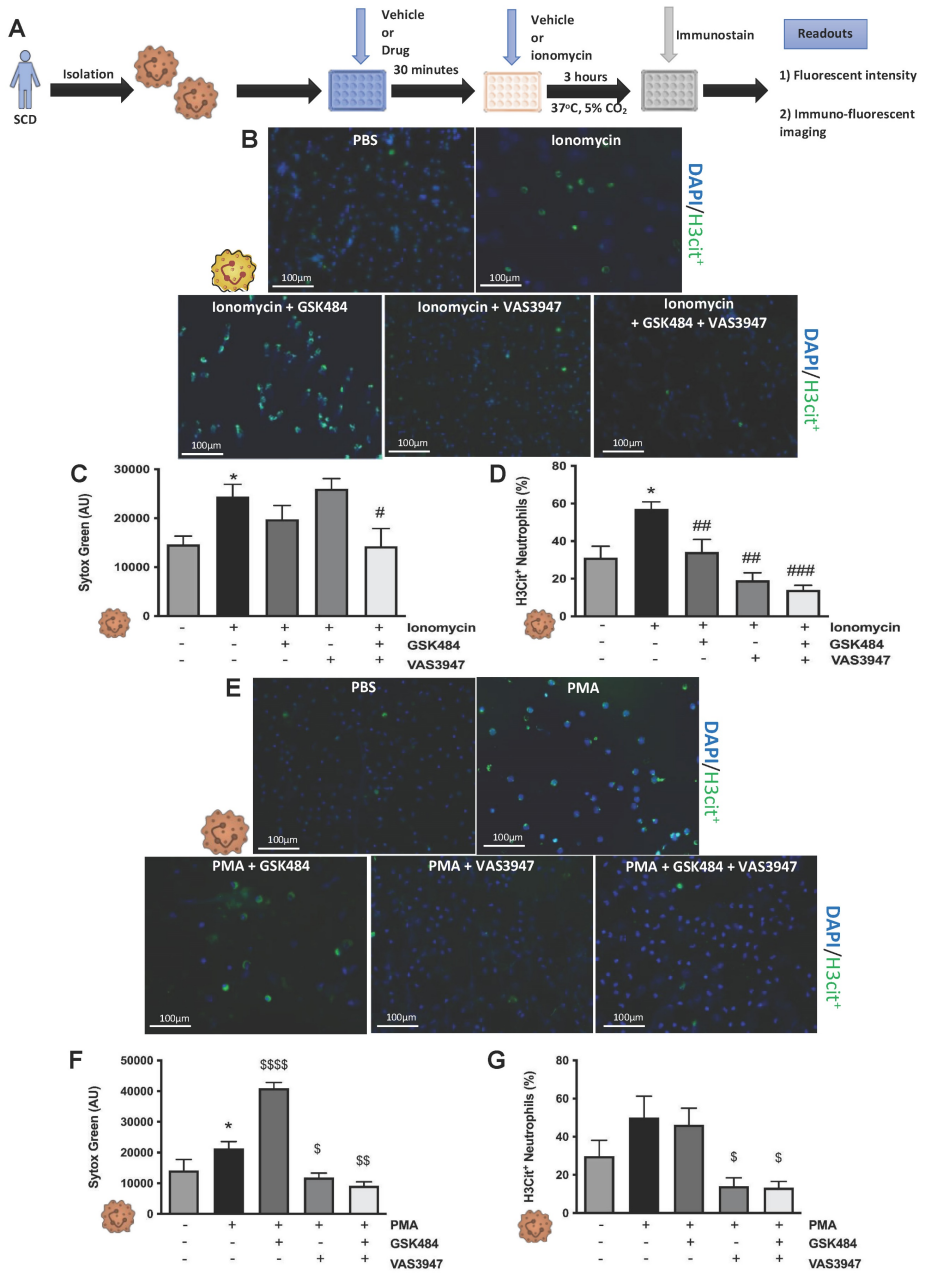
** Clinical thromboinflammation can be attenuated by targeting PAD4 and ROS signalling in human neutrophils. (A)** Schematic of experimental design of neutrophil isolation and neutrophil extracellular trap (NET) analysis. **(B+E)** Images of NETs with merged H3Cit (green/Alexa Fluor 488), NE and nucleus (Blue/49,6-diamidino-2-phenylindole). Bars in images represent 100 mm. **(C)** NETs were quantified by Sytox green intensity from unstimulated and ionomycin (4 µM)-stimulated neutrophils isolated from sickle cell disease (SCD) patients; unstimulated (n = 10), ionomycin stimulated (n = 10), GSK484 pre-treated ionomycin-stimulated SCD neutrophils (n=8), VAS3947 pre-treated ionomycin-stimulated SCD neutrophils (n=5), and GSK484 + VAS3947 pre-treated ionomycin-stimulated SCD neutrophils (n=5). **(D)** In a similar manner, percentage of H3Cit^+^ neutrophils were quantified from unstimulated (n = 8), ionomycin-stimulated (n = 8), GSK484 pre-treated ionomycin-stimulated SCD neutrophils (n=8), VAS3947 pre-treated ionomycin-stimulated SCD neutrophils (n=6), and GSK484 + VAS3947 pre-treated ionomycin-stimulated SCD neutrophils (n=6). **(F)** Further NETs were also quantified by Sytox green intensity from unstimulated and PMA (100 nM)-stimulated SCD neutrophils isolated from SCD patients; unstimulated (n = 5), PMA-stimulated (n = 5), GSK484 pre-treated ionomycin-stimulated SCD neutrophils (n=5), VAS3947 pre-treated ionomycin-stimulated SCD neutrophils (n = 5), and GSK484 + VAS3947 pre-treated ionomycin-stimulated SCD neutrophils (n = 5). **(G)** Percentage of H3Cit^+^ neutrophils were quantified from unstimulated (n = 8), PMA stimulated (n = 8), GSK484 pre-treated PMA-stimulated SCD neutrophils (n = 6), VAS3947 pre-treated PMA-stimulated SCD neutrophils (n = 5), and GSK484 + VAS3947 pre-treated PMA-stimulated SCD neutrophils (n = 5). ^*^p<0.05 vs. control unstimulated SCD neutrophils. ^#^*p<*0.05, ^##^*p<*0.01, ^###^*p<*0.001 vs. ionomycin-stimulated SCD neutrophils. ^$^*p<*0.05, ^$$^*p<*0.01, ^$$$$^*p<*0.001 PMA stimulated SCD neutrophils. Graphs are expressed as mean±SEM from independent experiments. All imaging analysis was done in a double-blinded fashion.

To build on our murine findings and translate them to a clinical setting, we next isolated neutrophils from SCD patients (**Figure 4**) and wanted to see whether the perturbation of PAD4 and NOX will result in attenuation of thromboinflammatory signalling. VAS3947 failed to inhibit ionomycin-induced extracellular DNA production (**Figure 4C**, n ≥ 5) but sufficiently inhibited ionomycin-induced histone citrullination in SCD neutrophils (**Figure 4B+D**, n ≥ 5). GSK484 failed to inhibit ionomycin-induced extracellular DNA production but was able to significantly attenuate ionomycin-induced histone citrullination in SCD neutrophils (**Figure 4B-D**). Combination of GSK484 and VAS3947 resulted in significant reduction of both ionomycin dependent histone citrullination as well as extracellular DNA production (**Figure 4B-D**). However, GSK484 failed to inhibit PMA dependent extracellular DNA production and histone citrullination in SCD neutrophils (**Figure 4F+G**, n ≥ 5). Finally, VAS3947 resulted in significant reduction of both extracellular DNA production and PMA-dependent histone citrullinetion (**Figure 4F+G**). All the above results further implicate PAD4 and ROS as an important target for inhibition of pathological NET production.

**Figure 5:**
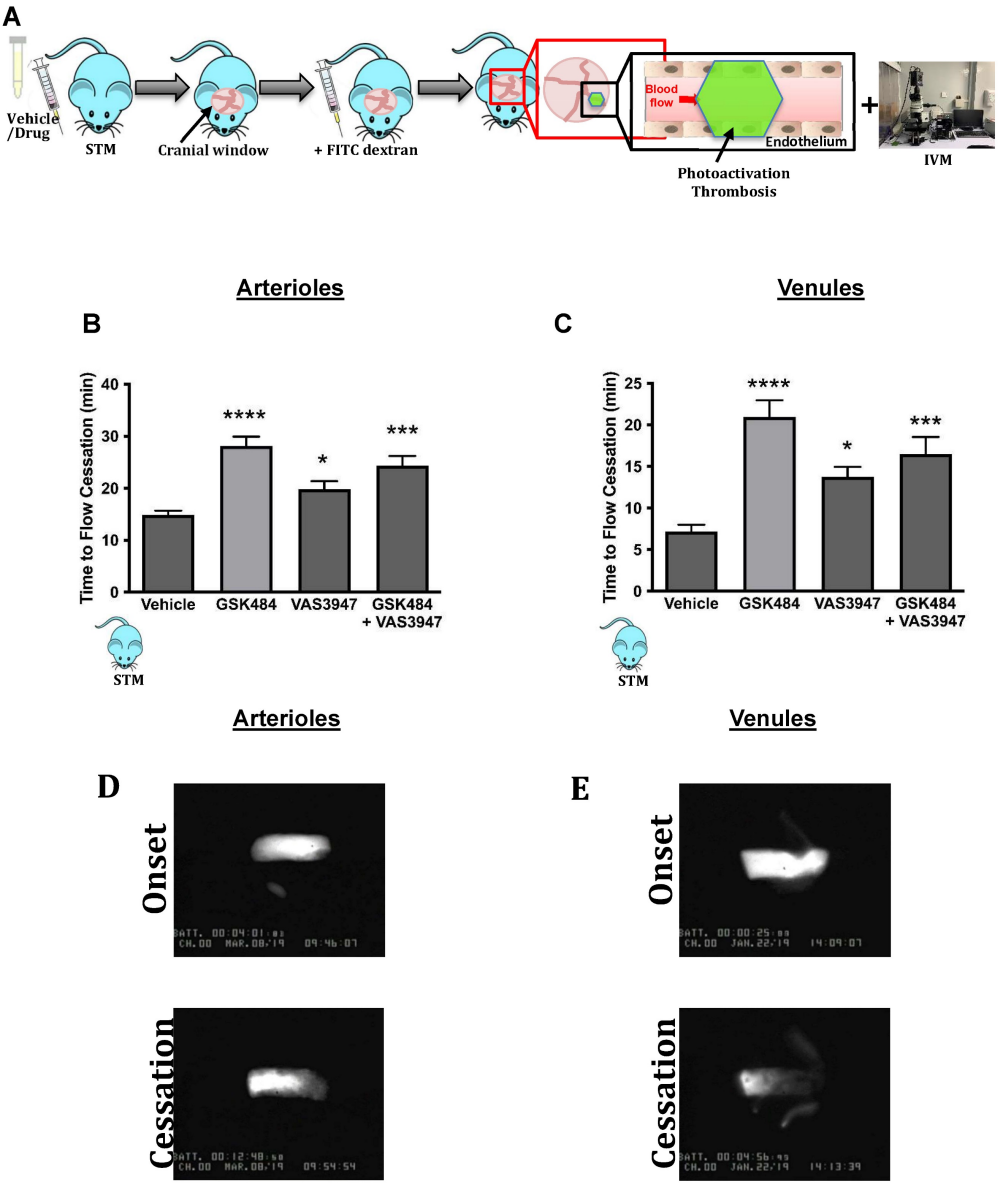
** Clinical thromboinflammation can be attenuated by targeting PAD4 and NOX *in-vivo*. (A)** Schematic representation of light/dye-induced thrombosis model induced with intravenous infusion of 10 mg/kg of 5% FITC-dextran followed by photoactivation of the cerebral microvessels of sickle cell transgenic mice (STM). Time to flow cessation (minutes) was defined as the complete stop of blood flow for ≥ 30 seconds and was assessed in **(B)** arterioles and **(C)** venules in STM treated with vehicle (saline. n=6 mice per group), GSK484 (10 μM, n=5 mice per group), VAS3847 (5 μM, n=5 mice per group) or a combination of GSK484 + VAS3947 (n = 5 mice per group). **(D-E)** Images of onset (start of platelet aggregation) and cessation (complete stop of flow for ≥30 seconds) in arterioles and venules respectively. ^*^p<0.05, ^**^p<0.01,^ ***^p<0.001, ^****^p<0.0001 vs. vehicle treated mice. Graphs are expressed as mean±SEM from 5-6 mice per group.

Finally, to further validate our findings from SCD neutrophils, we used a humanised transgenic mouse model of SCD coupled to intravital microscopy to study thrombosis in a pathophysiological setting (**Figure 5A**). Inhibition of PAD4 and NOX (alone or in combination) significantly increased blood flow cessation times in cerebral microvessels (arterioles as well as venules) in STM, hence replicating the effects seen in C57BL/6 mice (**Figure 5B+C**, n ≥ 5).

## Discussion

We present herein several novel key conceptual findings and the first direct evidence that targeting PAD4 and NOX (two key neutrophil enzymes involved in innate immunity and disease) attenuates murine cerebrovascular inflammation and modifies neutrophil behaviour in human neutrophils, including SCD, towards an anti-thromboinflammatory phenotype. GSK 484 fails to inhibit PMA dependent extracellular DNA and H3Cit^+^ neutrophil production due to inability to target NOX dependent NET production. Furthermore, our findings illustrate that NOX-dependent ROS and not PAD4 is most likely a final and a key determinant in pathological NET production as targeting NOX only results in significant attenuation of H3Cit^+^ neutrophil production independent of stimuli and clinical phenotype. However, targeting both these mechanisms individually and in combination result in attenuation of *in-vivo* cerebrovascular phenotype in control as well as STM phenotype suggesting that both have significant role in cerebrovascular pathogenesis.

Neutrophils are implicated in the pathogenesis of various inflammatory conditions including cardiovascular diseases (e.g. myocardial infarction and stroke) 2, 14, 43 and are the most abundant cell type in human blood with high concentration of PAD4 and ROS proteins 29. Furthermore, there has been a significant advancement in the understanding of how neutrophils carry out various inflammatory functions via production of NETs and ROS 4, 5. Such inflammatory responses within the vascular system can be replicated within an *in-vitro* system by treating neutrophils with various stimuli such as ionomycin and PMA which can significantly promote pro-inflammatory phenotype 44. Our results demonstrated that targeting neutrophil PAD4 as well as ROS resulted in modification of NET production towards a more anti-inflammatory/anti-thrombotic phenotype. However, ROS targeting did not result in adequate inhibition of extracellular DNA, suggesting ROS is not necessary for extracellular DNA production 45.

It is known that one of the key elements of pathological NET production is histone citrullination 46. Both ionomycin and PMA resulted in significant neutrophil histone hypercitrullination 44 in control as well as SCD neutrophils 31. Although, PAD4 targeting was able to attenuate ionomycin dependent H3Cit^+^ neutrophil production, it failed to suppress PMA dependent H3Cit^+^ neutrophil production. These results suggest that PMA stimulation either bypasses PAD4 activation, or it employs an accessory ROS dependent PAD4 independent pathway for H3Cit^+^ neutrophil production 44, 45. The importance of ROS targeting was further proven by significant inhibition of H3Cit^+^ neutrophil production after ROS targeting in ionomycin as well as PMA stimulated neutrophils. Ionomycin also resulted in minimal ROS production, which is likely explained by the fact that ionomycin causes NET production by engaging PAD4 complex, independent of NOX 44, 45. Unsurprisingly, PMA induced NET production and NOX inhibition resulted in significant ROS production suppression.

PAD4 activation and translocation is a critical step for NET production and dysregulated PAD4 activation is known to enhance inflammatory responses in various chronic disease such as rheumatoid arthritis and diabetes mellitus 20, 47-49. Hence targeting PAD4 has been employed as a reasonable strategy to mitigate chronic inflammation and thrombosis 20. Although PAD2 is known to potentially attenuate thromboinflammatory response but this seems to be more macrophage dependent and therefore was not assessed in our study 25. There is also limited evidence of PAD2 is key for NET production 26, 50. Furthermore, there is ample evidence surrounding the involvement of ROS in the pathogenesis of vascular inflammation 51, 52. We found that targeting both these mechanisms within a clinically relevant cerebral thrombosis model (photoactivation thrombosis model) 42, resulted in significant attenuation of baseline thrombosis index.

SCD provides an excellent disease paradigm to study accelerated thrombosis. PAD4 as well as NOX are known to provoke thrombosis in known clinical models of inflammation 20, 28. However, this has not been specifically studied in cerebral thromboinflammation. Targeting PAD4 as well as NOX resulted in significant attenuation of thrombus formation in cerebral microvessels, thereby providing a proof of principle and a potential viable therapeutic strategy to target thromboinflammation driven stroke pathogenesis 53. Lastly, there is evidence that NETs are involved in the pathogenesis of COVID-19 and future studies should investigate the role of targeting PAD4 in attenuating COVID-19 related vascular disease 11, 21, 22, 24.

## Conclusions

Our discovery that PAD4 and NOX-dependent ROS modifies neutrophil behaviour in thromboinflammatory settings provides *potential therapeutic* strategy and target(s) for drug discovery programs focussed on the treatment and management of thrombosis and inflammation, conditions which underpin many of today's global health challenges.

## Supplementary Material

Supplementary figures and table, video legends.

Supplementary video 1.

Supplementary video 2.

Supplementary video 3.

Supplementary video 4.

## Abbreviations

ANOVA: analysis of variance
DAPI: 4',6-diamidino-2-phenylindole
DHR: dihydrorhodamine
DMEM: Dulbecco's modified eagle medium
EDTA: 2,2',2'',2'''-(Ethane-1,2-diyldinitrilo)tetraacetic acid
PBS: phosphate-buffered saline
H3cit^+^: Citrullinated histone H3
NET: Neutrophil extracellular trap
PAD4: Peptidyl Arginine Deiminase 4
PMA: phorbol 12-myristate 13-acetate
ROS: reactive oxygen species
SEM: standard error of mean
NOX: NADPH oxidase
SCD: Sickle cell disease
STM: Sickle transgenic mice
