# Supplementary material for: Regulating Neutrophil PAD4/NOX-Dependent Cerebrovasular Thromboinflammation
Source: Int J Biol Sci. 2023 Jan 9;19(3):852–64. doi: 10.7150/ijbs.77434 (PMC9910005; doi:10.7150/ijbs.77434)
Supplement: Supplementary file 1 — Supplementary figures and table, video legends. [file ijbsv19p0852s1.pdf]

Supplemental Material

Regulating Neutrophil PAD4/NOX-Dependent Cerebrovascular Thromboinflammation

Junaid Ansari, MD[1] Shantel A. Vital, MS[2] Shreya Yadav, MS[3] Felicity N. E. Gavins, PhD[3*]

Affiliations:

[1]Department of Neurology, Louisiana State University Health Sciences Center-Shreveport, Shreveport, LA, 71130, USA

[2]Department of Molecular & Cellular Physiology, Louisiana State University Health Sciences Center-Shreveport, Shreveport, LA, 71130, USA

[3]Department of Life Sciences, Centre of Inflammation Research and Translational Medicine (CIRTM), Brunel University London, London. UB8 3PH, UK.

[*]**Corresponding author:**

Felicity N. E. Gavins

Department of Life Sciences

Centre of Inflammation Research and Translational Medicine (CIRTM)

Brunel University London

London

UB8 3PH, UK.

Tel: +44 (0)1895 267151

Email: felicity.gavins@brunel.ac.uk

Supplemental Figure 1.

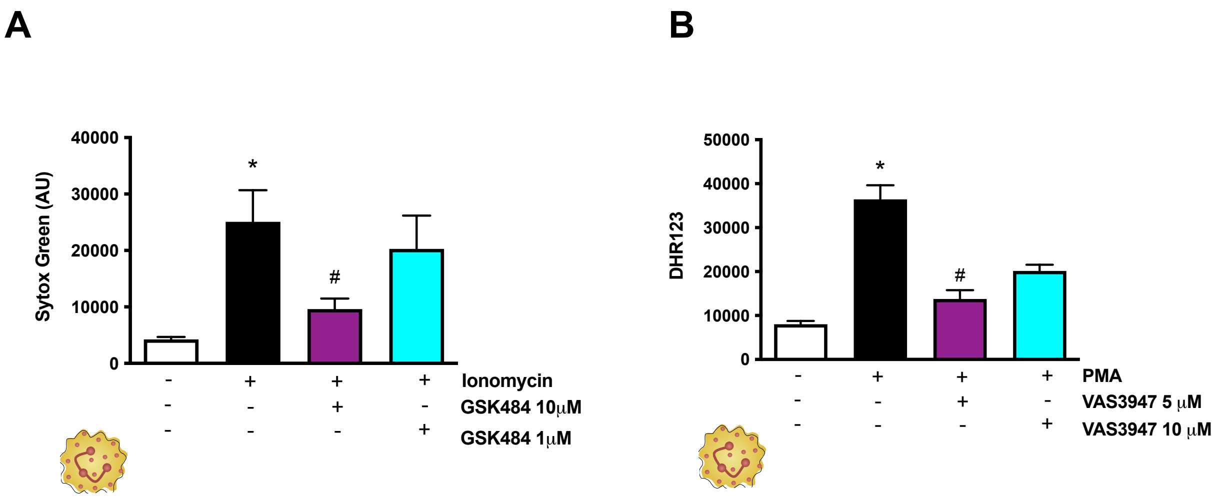

Supplemental Figure 1: GSK484 and VAS3947 dose response

(A) NETs were quantified by Sytox green intensity using a plate reader (BioTek; excitation = 485 nm, emission=525 nm) from unstimulated and ionomycin (4 μM)-stimulated neutrophils isolated from human volunteers; unstimulated (n=4), ionomycin stimulated (n = 4), GSK484 (10 μM) pre-treated ionomycin-stimulated neutrophils (n=4), and GSK484 (1 μM) pre-treated ionomycin-stimulated neutrophils (n=4) **(B)** DHR123 production was measured using a plate reader (BioTek; excitation = 485 nm, emission = 525 nm) from unstimulated neutrophils, PMA-stimulated neutrophils, VAS3947 (5 μM) treated PMA-stimulated neutrophils and VAS3947 (5 μM) treated PMA-stimulated neutrophils (n=3 in all groups). Graphs are expressed as mean±SEM from independent experiments. All imaging analysis was done in a double-blinded fashion.

Supplemental Figure 2.

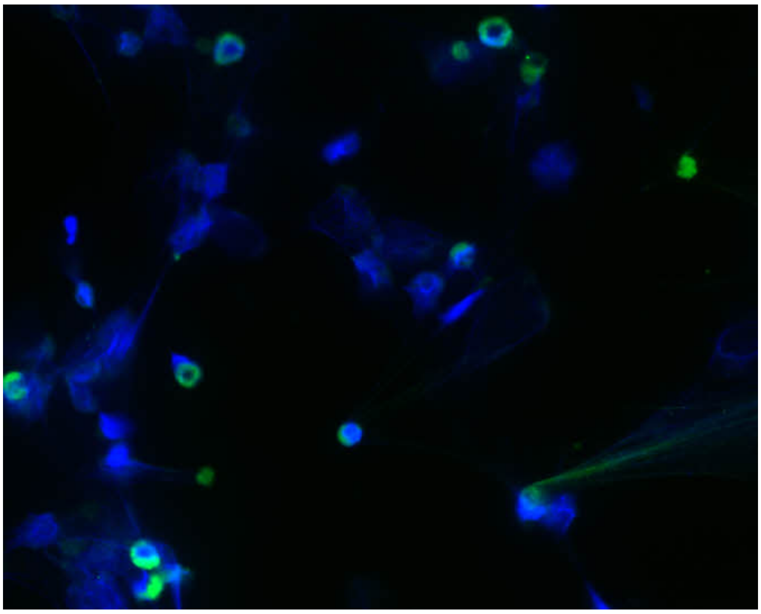

Supplemental Figure 2: For immunocytochemistry, cells were incubated with NET specific antibodies histone H3 mouse anti-H3Cit (1:200) followed by species-specific secondary antibodies coupled with Alexa Fluor Dyes (1:1000, Alexa Flour 488 goat anti-mouse, green) and co-stained with DAPI (nuclear stain, blue) After mounting (Fluoromont-G, Southern Biotech, Birmingham, Alabama, USA) the images were visualized by Nikon Eclipse Ti inverted epifluorescence microscope (Minato-ku, Tokyo, Japan). We quantified *in-vitro* NETs by measuring the percentage of CitH3 stained DNA over total number of cells (DAPI stained). Figure shows a protrusion or a thread like structure coming from the main cell body, which was positive for nuclear stain DAPI, and neutrophil elastase (NET bound protein), as well as citrullinated histone.

Supplemental Table 1. Demographic and clinical characteristics of controls and Sickle Cell Disease (SCD) patients

**Variable**	**Control**	**SCD patients**
Number	15	14
Gender	Eight males, seven females	Five males, nine females
Age range	25-54 years old	20-36 years old
History of cerebrovascular accidents	0	5 (38.46%)
Leukocytes (Ref: 3.6-11.2 K/µl)	Not recorded	10.73 ± 1.09
Red blood cells (Ref: 4.06-5.63 M/µl)	Not recorded	2.95 ± 0.19
Hemoglobin (Ref: 12.5-16.3 gm/dl)	Not recorded	9.12 ± 0.37
Hematocrit (Ref: 36.7-47.1 %)	Not recorded	26.84 ± 1.23
Platelet count (Ref: 159-386 K/µl)	Not recorded	310.6 ± 30.13
Neutrophils (Ref: 1.8-7.8 K/µl)	Not recorded	6.8 ± 0.90

'Ref:' indicates reference range. Data represented as mean ± SEM in SCD patients.

Video Legends

Video 1. Time-lapse video microscopy shows progression of thrombus formation in a cerebral arteriole of a control mouse demonstrating onset and complete cessation of blood flow.

Video 2. Time-lapse video microscopy shows progression of thrombus formation in a cerebral venule of a control mouse demonstrating onset and complete cessation of blood flow.

Video 3. Time-lapse video microscopy shows progression of thrombus formation in a cerebral arteriole of an STM mouse demonstrating onset and complete cessation of blood flow.

Video 4. Time-lapse video microscopy shows progression of thrombus formation in a cerebral venule of an STM mouse demonstrating onset and complete cessation of blood flow.
